# Adverse Events following 12 and 18 Month Vaccinations: a Population-Based, Self-Controlled Case Series Analysis

**DOI:** 10.1371/journal.pone.0027897

**Published:** 2011-12-12

**Authors:** Kumanan Wilson, Steven Hawken, Jeffrey C. Kwong, Shelley Deeks, Natasha S. Crowcroft, Carl Van Walraven, Beth K. Potter, Pranesh Chakraborty, Jennifer Keelan, Michael Pluscauskas, Doug Manuel

**Affiliations:** 1 Department of Medicine, Ottawa Hospital Research Institute, University of Ottawa, Ottawa, Canada; 2 ,Ottawa Hospital Research Institute, University of Ottawa, Ottawa, Canada 2ICES@Uottawa; 3 Department of Epidemiology and Community Medicine, University of Ottawa, Ottawa, Canada; 4 Newborn Screening Ontario, Children's Hospital of Eastern Ontario, Ottawa, Canada; 5 Institute for Clinical Evaluative Sciences, Toronto, Ontario, Canada; 6 Ontario Agency for Health Protection and Promotion, Toronto, Ontario, Canada; 7 Dalla Lana School of Public Health, University of Toronto, Toronto, Canada; 8 Department of Pediatrics, University of Ottawa, Ottawa, Canada; 9 Department of Family Medicine, University of Ottawa, Ottawa, Canada; University of Witwatersrand, South Africa

## Abstract

**Background:**

Live vaccines have distinct safety profiles, potentially causing systemic reactions one to 2 weeks after administration. In the province of Ontario, Canada, live MMR vaccine is currently recommended at age 12 months and 18 months.

**Methods:**

Using the self-controlled case series design we examined 271,495 12 month vaccinations and 184,312 18 month vaccinations to examine the relative incidence of the composite endpoint of emergency room visits or hospital admissions in consecutive one day intervals following vaccination. These were compared to a control period 20 to 28 days later. In a post-hoc analysis we examined the reasons for emergency room visits and the average acuity score at presentation for children during the at-risk period following the 12 month vaccine.

**Results:**

Four to 12 days post 12 month vaccination, children had a 1.33 (1.29–1.38) increased relative incidence of the combined endpoint compared to the control period, or at least one event during the risk interval for every 168 children vaccinated. Ten to 12 days post 18 month vaccination, the relative incidence was 1.25 (95%, 1.17–1.33) which represented at least one excess event for every 730 children vaccinated. The primary reason for increased events was statistically significant elevations in emergency room visits following all vaccinations. There were non-significant increases in hospital admissions. There were an additional 20 febrile seizures for every 100,000 vaccinated at 12 months.

**Conclusions:**

There are significantly elevated risks of primarily emergency room visits approximately one to two weeks following 12 and 18 month vaccination. Future studies should examine whether these events could be predicted or prevented.

## Introduction

The measles, mumps and rubella (MMR) have been used extensively in children and have been demonstrated to be safe and effective in preventing disease [1]. However, because it is a live vaccine the MMR vaccine has the potential to cause adverse events one to 2 weeks following vaccination [2]. Most reactions to this vaccine will be mild with fevers occurring in 5 to 15% and rashes in 5% [3]. More serious reactions are extremely rare and may not be identified during pre-licensure trials [4]. Post market surveillance has identified an incidence of febrile seizures following the MMR vaccine of 25 to 34 per 100 000 vaccinated and a two to three-fold increased relative risk [5], [6]. However, at a population level, mass exposures to a vaccine with a rare side effect profile could have detectable important population level effects. No study has examined the impact on aggregate health service utilization following the MMR vaccination.

In the province of Ontario, Canada, the MMR and meningococcal C vaccines are currently recommended at 12 months of age and a second dose of MMR vaccine along with a booster dose of pentavalent (diphtheria, acellular pertussis, tetanus, polio and *Haemophilus influenzae* type b) vaccine is recommended at 18 months of age. We sought to examine the population wide effects of these vaccinations on the combined endpoint of emergency room visits and hospital admissions in selected periods post-vaccination.

## Methods

### Design

The overall goal of this study was to determine the risk of serious adverse events in all children vaccinated in Ontario at 12 and 18 months of age with recommended pediatric vaccines. This was measured by comparing the risk of either presentation to emergency room (ER), or hospital admission in consecutive one day periods after the date of vaccination compared to a later control period. This analysis was conducted on all children born between April 1^st^ 2006 and March 31^st^ 2009. Our primary analysis of the composite risk of ER visits and hospitalizations was conducted using the *self-controlled case-series design*, described by Farrington and associates [7], [8]. We analyzed events following the 12 and 18 month vaccinations separately.

### Data

Our study cohort included all children in the Newborn Screening Ontario data set between April 1^st^ 2006 and March 31^st^ 2009. This database captures over 99% of Ontario births. Our exposure of interest, pediatric vaccination, was identified using the Ontario Health Insurance Plan (OHIP) database. We used codes for general vaccination, as, except for influenza, vaccine-specific codes are not available. To identify the 12 and 18 month vaccinations separately we identified vaccination occurring on exactly the respective due dates as well as vaccinations occurring up to 60 days after the respective date. To allow adequate follow-up after the 12 month vaccination, only vaccinated children born on or before December 31^st^ 2008 could be included in the analysis (N = 271,495 children). Likewise, only vaccinated children born on or before June 30^th^ 2008 could be included in the analysis of adverse events after the 18 month vaccination (N = 184,312 children). Only subjects with both vaccinations and events in the observation period contribute to the conditional self-controlled case series analysis, therefore infants with no ER visits or hospitalizations in close proximity to the vaccination were not included. If infants had more than one vaccination in the database during the two month target period the first vaccination was used as the index vaccination. If another vaccination occurred within the observation period (0 to 28 days after the index vaccination), or the infant died, then this individual was excluded from analysis (see Appendix S1).

The Canadian Institute for Health Information's (CIHI) Discharge Abstract Database (DAD) captures all hospital admissions, including children in both tertiary and community hospitals, and was used to ascertain hospital admission. CIHI's National Ambulatory Care Registration System (NACRS) was used to ascertain ER visits, the Canadian Triage and Acuity Score (CTAS) rating and the diagnosis made by the most responsible physician for the visit. The Registered Persons Database was used to ascertain cases of death. These datasets are housed at the Institute for Clinical Evaluative Sciences (ICES), and linkage between datasets was achieved using encrypted health card numbers as unique identifiers. The study was performed within ICES' status as a Prescribed Entity in Ontario's privacy legislation and Research Ethics Board approval was received at OHRI and ICES (Sunnybrook).

### Analysis

**Figure 1:**
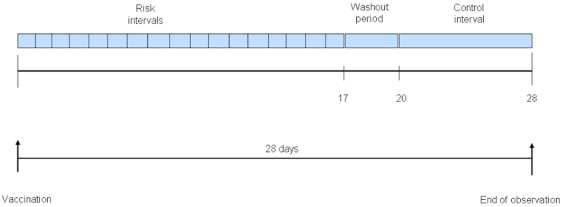
Illustration of the self-controlled case series design. The observation period for each patient begins with pediatric vaccination date (leftmost upward arrow) and continues for a total of 28 days. In the primary analyses, each day post vaccination is considered a *risk interval*, and consecutive days with a statistically significant t elevation in relative incidence were pooled to create a combined risk interval. Days 20–28 comprise the *control interval*. The intervening days represent the wash-out period.

We graphed the number of combined endpoint events in the days before and after vaccination. In the self-controlled case series model, the date of vaccination serves as the index date for exposure for each patient. Previous studies have identified that children are at increased risk for systemic reactions at different times from 5–14 days after vaccination [5], [6], [9], [10]. Because *a priori* we did not know with certainty the time period following vaccination for which there would be an increased risk of our combined endpoint, we modified the standard self-controlled case series approach by looking for an elevation in risk during each post-vaccination day up to day 17 (Figure 1). We then classified days 20–28 as unexposed, establishing a washout period in between the exposed and unexposed periods (Figure 1). When multiple events occurred to a given individual, the first occurrence of the composite outcome in the post-vaccination period was used (eg., someone attending the ER who was then admitted would have one event counted in that period). The relative incidence rate of the composite endpoint during the exposed period compared with the unexposed period was analyzed using a fixed effects Poisson regression model. This model included a term for exposure period and a term for patient, thereby allowing each individual to serve as his or her own control and accounting for intra-individual correlation. An offset term was also included to account for the differing durations of the exposed and unexposed periods. Deaths after the 12 and 18 month vaccinations were explored in a separate analysis due to the fact that a subject dying effectively truncates their follow-up potentially biasing the results of the SCCS analysis. As noted above, children who died during the follow-up period were excluded from the SCCS analysis of ER visits and hospitalizations.

To define the at-risk period we combined consecutive days with statistically significant elevations in relative incidence. We considered statistical significance to be a p-value less than or equal to 0.001 based on a Bonferroni correction to account for multiple testing (38 separate tests) [11]. We conducted separate analyses for the 12 and 18 month vaccinations. We also conducted secondary analyses to determine the association between vaccination and ER visits, hospital admissions, and deaths separately. All p values were 2 sided, and analyses were conducted using SAS version 9.2 (SAS Institute, Cary, NC).

In order to assess the types of cases captured by our endpoints we conducted a post-hoc analysis where we compiled the reasons for presentation to the ER as determined by the most responsible physician for the risk period for the 12 month vaccination. This was compared to the prevalence of the same diagnoses in the control period. We examined a tracer condition, ear/face nose injury, for which we do not expect a difference in rates. We also identified the CTAS ratings for presentations during the affected period and compared them to those during the control period using the Wilcoxon Rank-Sum test. CTAS ratings range from 1 to 5 with 1 representing a severe condition requiring resuscitation and 5 representing a less severe condition requiring non-urgent care [12]. In another post-hoc analysis we graphically examined the pattern of events following 12 and 18 month vaccination in the years 2002–2005 when the MMR vaccine was still given at 12 months, however, the booster was given at five years and not eighteen months.

## Results

**Figure 2:**
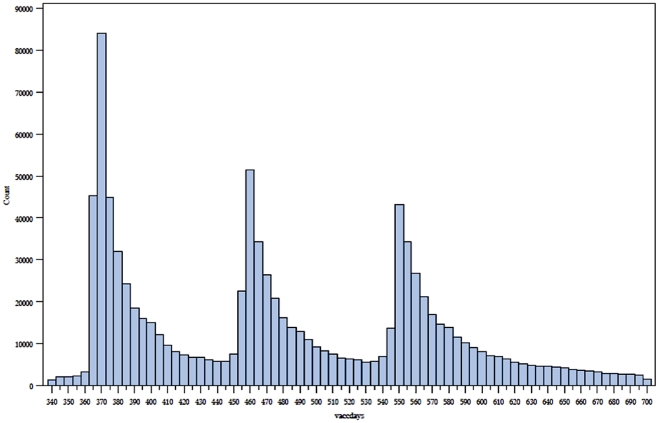
Vaccination events by days since birth from days 340 to 700. **Count** = number of individuals vaccinated on a given day. **Days** = number of days after date of birth.

**Figure 3:**
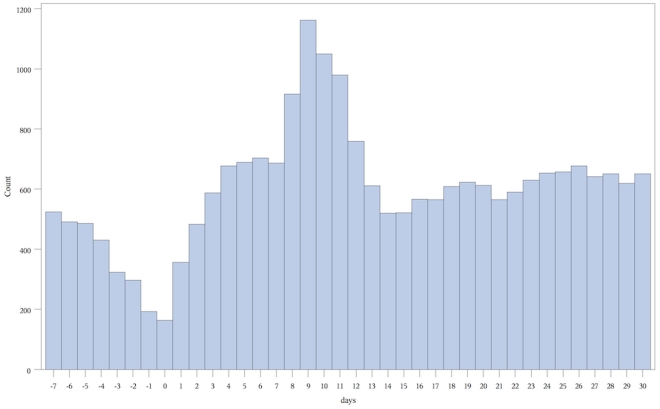
Number of combined endpoints versus days before/after 12 month vaccination. **Count** = number of combined endpoints of emergency room visit or hospitalization. **Days** = number of days before or after vaccination, day 0 being the day of vaccination.

**Figure 4:**
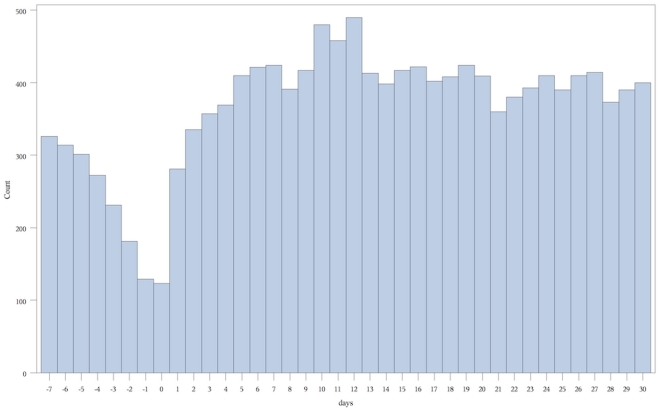
Number of combined endpoints versus days before/after 18 month vaccination. **Count** = number of combined endpoints of emergency room visit or hospitalization. **Days** = number of days before or after vaccination, day 0 being the day of vaccination.

In total, we examined 455,807 separate vaccination events in these 413,957 children that occurred at 12 and 18 months plus 60 days (Figure 2). We present the number of endpoint events versus days pre and post vaccination graphically for each of the vaccine periods (Figures 3 and 4).

### 12 month analysis

**Figure 5:**
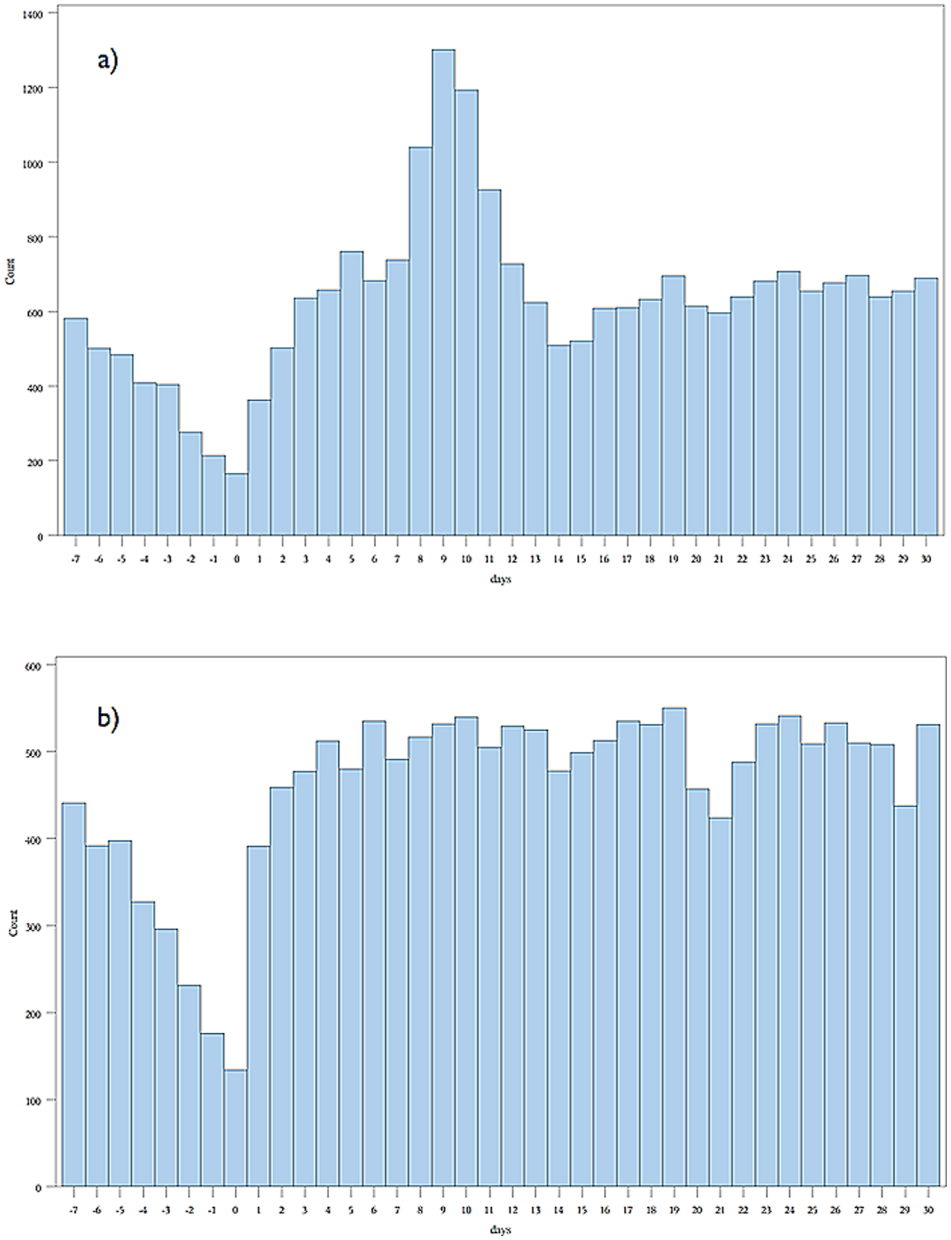
Historical analysis of combined endpoints versus days following 12 and 18 month vaccination: April 2002–March 2005. a) Before/after 12 month vaccination. b) Before/after 18 month vaccination. **Count** = number of combined endpoints of emergency room visit or hospitalization. **Days** = number of days before or after vaccination, day 0 being the day of vaccination.

271,495 children received vaccinations between 365 and 425 days of age. Consecutive statistically significant elevations in combined endpoints began on day 4 and continued to day 12. A total of 6462 children experienced at least one of the combined endpoints during the combined 9 day at risk period compared to 4845 during the 9 day control period. The relative incidence of the combined endpoint was 1.33 (1.29–1.38) (Table 1). The highest relative incidence during the at-risk period occurred between days 8 and 11 peaking at 2.04 (1.91–2.17) on day 9. Overall, an excess of 595 children experienced at least one of the combined endpoints during the risk interval per 100,000 vaccinated, or one additional child experiencing at least one endpoint during the risk interval for every 168 children who received their 12 month vaccinations (Table 2). Examining the historical graph of the events post 12 month vaccination in the years 2002–2005 demonstrated a similar peak in events (Figure 5).

**Table 1:** Relative incidence of combined endpoint (hospital admission or emergency room visit) following 12 month vaccination.

Risk interval*	Endpoints during risk interval (n)	Relative Incidence (95% CI)	P value
Day 4	621	1.15 (1.06–1.25)	0.0008
Day 5	641	1.19 (1.10–1.29)	<0.0001
Day 6	647	1.20 (1.11–1.31)	<0.0001
Day 7	644	1.20 (1.10–1.30)	<0.0001
Day 8	870	1.62 (1.50–1.74)	<0.0001
Day 9	1096	2.04 (1.91–2.17)	<0.0001
Day 10	991	1.84 (1.72–1.97)	<0.0001
Day 11	923	1.72 (1.60–1.84))	<0.0001
Day 12	713	1.32 (1.22–1.43)	<0.0001
Days 4 to 12** (Combined risk interval)	6462	1.33(1.29–1.38)	<0.0001
Days 20–28 (Control Interval)	4845	NA	NA

*Risk and control intervals expressed as days following vaccination.

**Total number of endpoints in the combined risk interval are less than the cumulative individual day event total because some children may have experienced events in multiple days and only the first event is counted.

**Table 2:** Increased risk of combined endpoints from vaccination.

Vaccination	Additional children experiencing at least one event (per 100,000 vaccinations)	Number vaccinated	Number vaccinated per excess event
12 months	595	271,495	168
18 months	137	184,312	730

The primary reason for the elevation in the combined endpoint was an increase in ER visits (relative incidence 1.34(1.29–1.39)). There were an excess of 598 children experiencing 1 or more ER visits during the risk interval per 100,000 vaccinations or 1 additional child for every 168 children vaccinated. There was no increase in hospital admissions (relative incidence 1.08 (0.93–1.25)). There were five or fewer deaths (Table 3). The average CTAS score for ER visits during the risk period was 3.27 compared to 3.26 for the control period. (p = 0.74), suggesting no differences in severity of presentation between ER visits in the risk and control periods. There was an increase in presentation for multiple conditions during the risk period compared to the control period. The largest relative risk was associated with febrile seizures (relative incidence = 2.34, fever (RI = 2.31) and viral exanthem (RI = 2.23). We calculated that there were approximately 20 additional febrile seizures during the risk interval for every 100 000 children vaccinated. There was no increase in our tracer condition (ear/face/nose injury).

**Table 3:** Relative incidences of individual endpoints (emergency room visit, hospital admission, death) during highest risk interval compared to control period.

Outcome	12 months	Events (risk/control)	18 months	Events (risk/control)
Emergency visits	1.34 (1.29–1.39)	6395/4772	1.25 (1.18–1.34)	1264/3024
Admissions	1.08 (0.93–1.25)	356/330	1.23 (0.94–1.59)	78/191
Deaths	-	< = 5/< = 5	-	0/0

### 18 month analysis

184,312 children received vaccinations between 545 and 605 days of age. Consecutive statistically significant elevations in combined endpoints began on day 10 and continued to day 12. A total of 1275 children experienced at least one event included in the combined endpoint during the combined three day at risk period compared to 3065 during the nine day control period. The relative incidence of the combined endpoint was 1.25 (1.17–1.33) (Table 4). The highest relative incidence during the at-risk period was 1.34 (1.21–1.47) which occurred on day 12. Overall, an additional 137 children experienced at least one combined endpoint during the three day risk period per 100,000 vaccinated, or one additional child experiencing at least one excess event for every 730 children vaccinated (Table 3). Examining the historical graph of the events post 18 month vaccination in the years 2002–2005, when the booster dose of the MMR vaccine was not given, demonstrated no similar peak in events (Figure 5).

**Table 4:** Relative incidence of combined endpoint (hospital admission or emergency room visit) following 18 month vaccination.

Risk interval*	Endpoints during risk interval (n)	Relative Incidence(95% CI)	P value
Days 10	447	1.31 (1.19–1.45)	<0.0001
Days 11	428	1.26 (1.14–1.39)	<0.0001
Days 12	455	1.34 (1.21–1.47)	<0.0001
Days 10 to 12 (Combined risk interval)	1275	1.25 (1.17,1.33)	<0.0001
Days 20 to 28 (Control Interval)	3065	NA	NA

*Risk and control intervals expressed as days following vaccination.

The primary reason for the elevation in the combined endpoint was an increase in ER visits (relative incidence 1.25(1.18–1.34)). There were an excess of 139 children experiencing one or more ER visits during the risk interval or one excess visit for every 719 children vaccinated. There was not a significant increase in hospital admissions (relative incidence 1.23(0.94–1.59)) (Table 4). No deaths occurred in the risk or control periods.

## Discussion

Our analysis demonstrated that the 12 and 18 month vaccinations are not associated with an increase in adverse events immediately following vaccination. Instead it showed a reduced risk in this period, which is likely a result of the previously documented healthy vaccinee effect [9], [13], [14]. We identified an increase in events occurring between 4 and 12 days post-vaccination for the 12 month and, to a lesser extent and for a shorter time period for the 18 month vaccines. The majority of these events represented ER visits and at their peak, on day 9 following the 12 month vaccine, were approximately twice the baseline rate. Although there was an increase in hospital admission in each period, none of these increases were statistically significant. Overall the increase in event rate following the 12 month vaccines accounted for approximately 598 extra children experiencing one or more ER visits during the risk interval per 100,000 vaccinations. The average acuity of patients presenting to the emergency room was similar to that in the control period. The conditions for which there were the largest increase in risk for presentation to the emergency room during the risk interval compared to the control interval following the 12 month vaccine were febrile convulsions, fever and viral exanthema, consistent with the known adverse event profile of MMR and varicella vaccines. There were 20 additional febrile seizures for every 100,000 children vaccinated at 12 months.

The development of an inflammatory response approximately one week after vaccination is recognized in the literature. For example, the Centres for Disease Control and Prevention list days 7 to 12 post vaccination as the highest risk period for developing fever and possibly a rash [15]. This closely coincides with our observation of the time period during which emergency room visits peaked. A previous twin study also identified the development of systemic symptoms between days 6 and 14 and peaking on day 10 [9]. A study of febrile seizures following MMR vaccination identified the highest at risk period to be 8 to 14 days following vaccination and a relative risk of 2.83 and other studies have made similar observations [5], [6], [16]. These are consistent with our findings. While it is known that vaccines can produce these adverse events, our study demonstrated the population wide impact of this effect and that these events are resulting in an increase in health services utilization. The estimated 595 additional children experiencing at least one event for every 100 000 vaccinated translates into approximately one child experiencing at least one event per 168 children vaccinated. The explanation for this effect is likely the controlled replication of the virus creating a mild form of the illness the vaccine is designed to prevent. The top diagnoses for the presentations to the emergency room during the 12 month risk interval would all be consistent with a mild viral illness.

The reduced effect at 18 months is likely due to this vaccination in most instances being a second exposure to the antigen to which the vast majority of children would have developed adequate immunity. Residual events during this period may represent the small percentage of children who did not immunologically respond to the first dose of the vaccine.

Our study has several strengths. The use of the self-controlled case series design allows for individuals to serve as their own controls implicitly controlling for all fixed covariates [8], [17]. Seasonal confounding is unlikely to have influenced our findings since the 12 and 18^th^ month vaccines are provided throughout the year. The potential for confounding due to co-existent exposures at 12 and 18 months exists, however, if such an exposure were to be significant we would have expected to observe an effect at 18 months in our historical analysis. Our study included nearly all children born in Ontario during the study period which strengthens the generalizability of these findings. The combination of the self-controlled case series design and our sample size increased the power of our study to identify small effects. While our study cannot establish causality it has many features that support a causal relationship between vaccination and delayed adverse events. These include the consistency with other studies and a compelling biological model which explains the diagnoses in the affected children and the reduction in effect with the 18 month vaccinations. Furthermore, our historical analysis demonstrates that the effect seen at 18 months after MMR vaccination in 2006–2009 is not present in 2002–2005, when the MMR vaccine was given only at 12 months and not at 18 months. The effect is still clearly visible after the 12 month vaccination in the 2002–2005 data.

There are important limitations of this study. The first is that, as mentioned, the healthy vacinee effect may have masked an association in the immediate post-vaccination period. Second, we cannot know whether a specific vaccine was associated with the adverse events as multiple vaccines are typically administered at each visit. However, we have previously demonstrated the safety of the pentavalent vaccine which is given with the 18 month MMR vaccine [18]. It is possible that the effects seen at 12 month are in part due to the potential co-administration of the meningococcal C vaccine, however, this is not a live vaccine and should create inflammation in the immediate post-vaccination period as opposed to one week later. Third, the codes we used for identifying the reasons for presentation to the emergency room have not been validated. However, we would expect that the diagnoses of febrile convulsion to have a low misclassification error and has previously been validated as a useful ER code in a separate dataset [19]. We also did not look for increases in visits to physician offices that did not result in presentation to the emergency room or admission and cannot comment on the impact of immunization on that outcome.

Our findings have important implications for those providing care to children. The immediate risk of a serious adverse event following immunization is low with both the vaccination visits that contain the MMR and varicella vaccines. However, the 12 month vaccines which typically contain the first dose of the MMR vaccine is associated with an increased risk of an emergency room visit approximately 4 to 12 days after immunization, peaking between days 8 and 11. This increase in rate of a child experiencing at least one event for every 158 vaccinated individuals is associated with a similar acuity as the control period. If the presentation to the emergency room was due to parental anxiety we would have expected to see a reduction in acuity during the risk period. The findings also suggest that the reactions are not severe since acuity was not higher than the control period and furthermore, there were few hospital admissions. Additional reassurance can be derived from previous studies that identified no long-term consequences related to vaccine associated febrile seizures [5], [6]. The increase in ER visits we observed could be a result of insufficient information being provided to parents who may not expect their child to develop a reaction a week after vaccination. In particular, the likelihood of this risk may be underestimated by physicians. Our study also reinforces the reduced risk of events following the second dose of MMR vaccine.

Given the effectiveness of the MMR vaccine in eliminating both measles and rubella, and the highly infectious nature of these diseases, high vaccination coverage is essential. The diseases that the vaccines are preventing are not benign and vaccination can eliminate many of the serious sequelae of these infections [20]. Complications from measles include otitis media (7–9% of cases), pneumonia (1–6% of cases), encephalitis (1 per 1,000–2,000 cases), subacute sclerosing panecephalitis (1 per 100,000 cases), and death (1 per 3000 cases) [3], [21]. Further studies attempting to predict which children develop post-vaccination reactions, as well as determining the effectiveness of prophylactic treatment with antipyrectics prior to the high risk period for symptom development are warranted.

## Supporting Information

Appendix S1 Figure A1: Flowchart Describing SCCS Study Cohort.
(TIF)
