# Supplementary figures and images for: Adverse Events following 12 and 18 Month Vaccinations: a Population-Based, Self-Controlled Case Series Analysis
Source: PLoS One. 2011 Dec 12;6(12):e27897. doi: 10.1371/journal.pone.0027897 (PMC3236196; doi:10.1371/journal.pone.0027897)

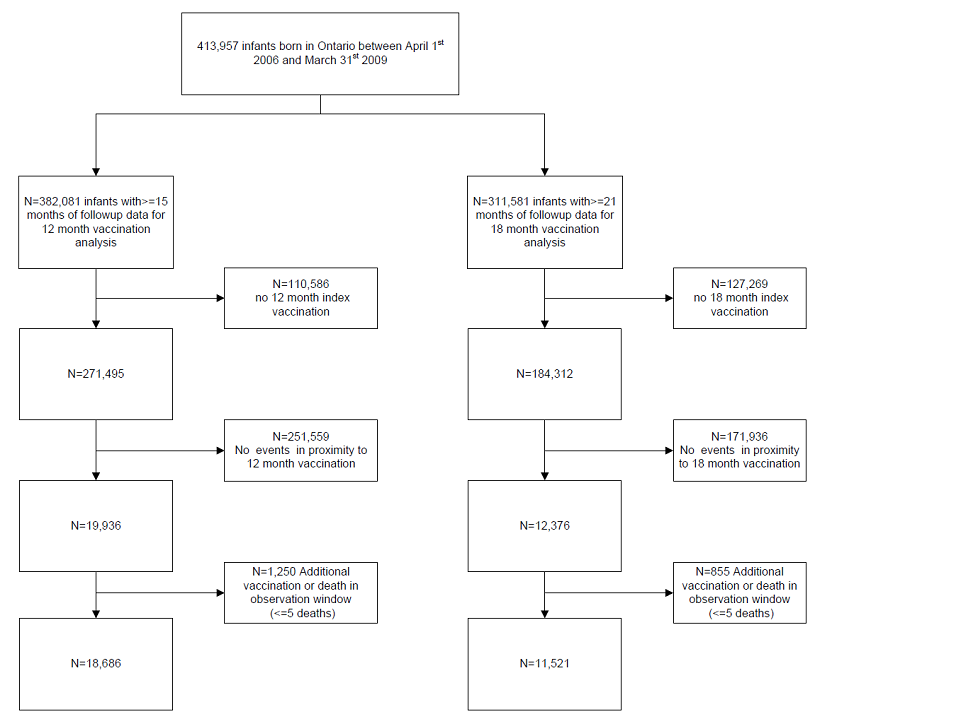

Supplement: Appendix S1 — Figure A1: Flowchart Describing SCCS Study Cohort. (TIF) [file pone.0027897.s001.tif]
